# Associations between abuse/neglect and ADHD from childhood to young adulthood: A prospective nationally-representative twin study

**DOI:** 10.1016/j.chiabu.2018.04.025

**Published:** 2018-07

**Authors:** Adi Stern, Jessica Agnew-Blais, Andrea Danese, Helen L. Fisher, Sara R. Jaffee, Timothy Matthews, Guilherme V. Polanczyk, Louise Arseneault

**Affiliations:** a Social, Genetic, and Developmental Psychiatry Centre, Institute of Psychiatry, Psychology and Neuroscience, King’s College London, London, United Kingdom; b Department of Psychology, University of Pennsylvania, Philadelphia, PA, USA; c University of São Paulo, São Paulo, Brazil

**Keywords:** Maltreatment, Abuse/neglect, Childhood ADHD, Young adult ADHD, Conduct disorder, Longitudinal design

## Abstract

Child maltreatment has consistently been found to be associated with attention deficit/hyperactivity disorder (ADHD). However, the robustness of this association and the direction of the link between maltreatment and ADHD remain unclear. We used data from the Environmental Risk (E-Risk) Longitudinal Twin Study, a cohort of 2232 British twins, to investigate the associations between exposure to abuse/neglect and ADHD in childhood and in young adulthood, and to test their robustness and specificity. We also aimed to test longitudinal associations between abuse/neglect and ADHD from childhood to young adulthood, controlling for confounders. Results indicated strong associations between abuse/neglect and ADHD in childhood and also in young adulthood. In childhood, the association was concentrated among children with comorbid conduct disorder. Longitudinal analyses showed that childhood ADHD predicted abuse/neglect in later years. This association was again concentrated among individuals with comorbid conduct disorder. Abuse/neglect in childhood was not associated with later ADHD in young adulthood after adjusting for childhood ADHD. Our study does not provide support of a causal link between child abuse/neglect and adult ADHD but highlights the possibility of a long-term effect of disruptive behaviors on the risk for experiencing abuse/neglect. These findings emphasize the need for clinicians treating people with ADHD, especially those with comorbid conduct disorder, to be aware of their increased risk for experiencing abuse/neglect. Interventions aimed at reducing risks of abuse/neglect should also focus on the environment of individuals with disruptive behaviors.

## 1 Introduction

Childhood maltreatment including abuse (physical, emotional and sexual) and neglect (physical and emotional) can affect between 2.5–32% of children worldwide (Afifi et al., 2014; Gilbert et al., 2009; Radford et al., 2011; Radford, Corral, Bradley, & Fisher, 2013) and is an important risk factor for the development of internalising and externalising psychopathology in adolescence and adulthood (Gilbert et al., 2009; Jaffee, Caspi, Moffitt, & Taylor, 2004; Kessler et al., 2010; Shonkoff, Garner, The committee on psychosocial aspects of child and family health, Committee on early childhood, adoption, and dependent care, & Section on developmental and behavioral pediatrics, 2012). Being a victim of maltreatment at a young age is related to symptoms of psychiatric disorders (e.g., depression, anxiety, and post-traumatic stress disorder) in later years, as well as to alcohol and cannabis abuse, antisocial behavior and conduct disorder (Afifi et al., 2014; Cecil, Viding, Barker, Guiney, & McCrory, 2014; Fisher et al., 2010; Gilbert et al., 2009; Newbury et al., 2018; Widom, DuMont, & Czaja, 2007; Widom, White, Czaja, & Marmorstein, 2007). However, many challenges remain for establishing causal relationships between child maltreatment and mental health problems. We focused on clarifying the nature of the association between child maltreatment and attention deficit/hyperactivity disorder (ADHD).

ADHD is characterized by a persistent pattern of inattention and/or hyperactivity-impulsivity that interferes with functioning and development (Diagnostic and Statistical Manual of Mental Disorders, 5th edition; [DSM-5]; American Psychiatric Association (APA), 2013). It is one of the most common neurodevelopmental disorders in childhood, with an estimated prevalence of 3.4% (Polanczyk, Salum, Sugaya, Caye, & Rohde, 2015). Childhood ADHD has been associated with poor functional outcomes (Larson, Russ, Kahn, & Halfon, 2011) and comorbid psychiatric disorders including oppositional defiant, conduct and learning disorders (Jensen & Steinhausen, 2015; Thapar & Cooper, 2016; Thapar, Cooper, Eyre, & Langley, 2013). ADHD is highly heritable in childhood with genetic factors explaining 60–90% of the variance (Faraone & Mick, 2010; Larsson, Chang, D’Onofrio, & Lichtenstein, 2014; Thapar & Cooper, 2016; Thapar et al., 2013).

Once considered only a childhood disorder, ADHD is now recognized to persist and also emerge in adulthood (Agnew-Blais et al., 2016; Caye et al., 2016; Faraone, Biederman, & Mick, 2006; Moffitt et al., 2015). The estimated prevalence of adult ADHD ranges between 2.5% and 5% (Franke et al., 2012; Simon, Czobor, Bálint, Mészáros, & Bitter, 2009). Similar to children with ADHD, adults affected by ADHD experience poor functional outcomes (Barkley & Murphy, 2010; Erskine et al., 2016). Comorbid disorders among adults with ADHD include anxiety disorders, depression, substance use disorders, antisocial and other personality disorders (Erskine et al., 2016; Klassen, Katzman, & Chokka, 2010). Studies have indicated that the heritability in adulthood is lower than in childhood, accounting for approximately 30–41% of the variance of adult ADHD (Agnew-Blais et al., 2016; Franke et al., 2012).

Various forms of maltreatment have been associated with ADHD in children samples (Briscoe-Smith & Hinshaw, 2006; Dinkler et al., 2017; Endo, Sugiyama, & Someya, 2006; Gul & Gurkan, 2016; Hadianfard, 2014; Ouyang, Fang, Mercy, Perou, & Grosse, 2008; Sari Gokten, Saday Duman, Soylu, & Uzun, 2016). Similar findings were observed in adult samples: associations between retrospective reports of child maltreatment and adult ADHD have been reported (Capusan et al., 2016; Fuller-Thomson & Lewis, 2015; Fuller-Thomson, Mehta, & Valeo, 2014; Rucklidge, Brown, Crawford, & Kaplan, 2006; Sanderud, Murphy, & Elkit, 2016; Singer, Humphreys, & Lee, 2016; Sugaya et al., 2012). Altogether, these studies indicate that maltreatment occurring prior to young adulthood is more common among people with ADHD compared to non-ADHD groups, and higher levels of ADHD symptoms are observed among individuals who were exposed to child maltreatment compared to non-exposed individuals. Yet, no study thus far has examined the association between ADHD and maltreatment in adolescent years separately from childhood. Adolescence is a time of major emotional, physical, social and neurodevelopmental change, suggesting that victimization during this period could have important implications for development (Fisher et al., 2015). Moreover, as adolescents spend an increasing proportion of their time outside the home environment, they are likely to experience a greater variety of types of victimization which could be associated to their ADHD symptoms. Most importantly, however, the robustness of this association (both in childhood and in young adulthood) and the direction of the link between maltreatment and ADHD have yet to be tested.

One study based on a large population-based sample of adult twins reported an association between child maltreatment and adult ADHD symptoms among monozygotic (MZ) twin pairs discordant for maltreatment (Capusan et al., 2016). The discordant MZ twin design tests whether twins exposed to maltreatment have more ADHD symptoms compared to their genetically-identical twin who was not exposed to maltreatment. Since the twins in this study grew up together, familial confounding factors were also controlled. Findings indicated that the association between ADHD and maltreatment within the MZ group was significant. Because of the stringent control for potential confounders, this study concluded that the association between child maltreatment and adult ADHD is partly causal. However, the validity of retrospective reports of childhood maltreatment has been questioned in light of possible misclassiﬁcation and bias (Reuben et al., 2016). In addition, it is necessary to consider temporal priority between the exposure and the outcome, requiring prospective population-based samples of children followed into adult years (Fuller-Thomson & Lewis, 2015). This is required because ADHD can be the result of maltreatment in childhood but can also be an early risk factor for experiencing maltreatment and other forms of violence victimization. Behavioral characteristics associated with ADHD, including being impulsive, making careless mistakes and interrupting or intruding on others, may evoke negative responses from the environment and produce or increase conflicts (Gul & Gurkan, 2016; Ouyang et al., 2008; Rucklidge et al., 2006; Sari Gokten et al., 2016).

In the present study, we used prospectively-collected measures from a longitudinal cohort study of twins to examine the association between exposure to abuse/neglect (including physical and sexual abuse, emotional abuse and neglect, and physical neglect) in childhood and adolescence, with ADHD up to age 12 and at age 18. First, we examined the associations between abuse/neglect and ADHD diagnoses in childhood and in young adulthood separately. We tested the robustness of these associations by also analysing ADHD symptom scales and by controlling for potential confounders. We also explored the specificity of these associations by looking at bullying and domestic violence. We further examined whether the association was concentrated specifically among ADHD participants with comorbid conduct disorder. In addition, we investigated twins’ differences in abuse/neglect and ADHD to control for familial confounding. Second, we investigated the longitudinal associations between abuse/neglect and ADHD from childhood into young adulthood.

## 2 Methods

### 2.1 Participants

Participants were members of the Environmental Risk (E-Risk) Longitudinal Twin Study, which tracks the development of a birth cohort of 2232 British children. The sample was drawn from a larger birth register of twins born in England and Wales in 1994–1995 (Trouton, Spinath, & Plomin, 2002). Full details about the sample are reported elsewhere (Moffitt & E-Risk Study team, 2002). Briefly, the E-Risk sample was constructed in 1999–2000, when 1116 families (93% of those eligible) with same-sex 5-year-old twins participated in home-visit assessments. This sample comprised 56% MZ and 44% dizygotic (DZ) twin pairs; sex was evenly distributed within zygosity (49% male). Families were recruited to represent the UK population with newborns in the 1990s, on the basis of residential location throughout England and Wales and mother’s age. Teenaged mothers with twins were over-selected to replace high-risk families who were selectively lost to the register through non-response. Older mothers having twins via assisted reproduction were under-selected to avoid an excess of well-educated older mothers. At follow-up, the study sample represents the full range of socioeconomic conditions in the United Kingdom (Odgers et al., 2012).

Follow-up home visits were conducted when children were aged 7 (98% participation), 10 (96%), 12 (96%), and 18 years (93%). Home visits at ages 5, 7, 10, and 12 included assessments with participants and their mothers (or primary caretaker). With parents’ permission, questionnaires were mailed to the children’s teachers, who returned questionnaires for 94% of children at age 5 years, 93% of those followed up at age 7 years, 90% at age 10 years, and 83% at age 12 years. The home visits at age 18 included interviews only with participants (n = 2066). There were no significant differences between those who did and did not take part at age 18 years in socioeconomic status when the cohort was initially defined (χ^2^ = 0.86; p = 0.65), age-5 IQ scores (t = 0.98; p = 0.33), age-5 behavioral or emotional problems (t = 0.40; p = 0.69 and t = 0.41; p = 0.68, respectively), or rates of childhood ADHD (χ^2^ = 2.08; p = 0.72). At age 18 years, participants were asked to identify two individuals who know them well to act as co-informants; 99.3% of participants had co-informant data.

The Joint South London and Maudsley and the Institute of Psychiatry Research Ethics Committee approved each phase of the study. Parents gave written informed consent and twins gave assent between ages 5 and 12 and then written informed consent at age 18. Analyses in this paper were restricted to 2040 individuals with ADHD information in childhood and in adulthood.

### 2.2 Measures

#### 2.2.1 Victimization

##### 2.2.1.1 Childhood victimization

The measurement of childhood victimization has been described previously (Danese et al., 2017; Schaefer et al., 2017; details are provided in the Supplementary materials). Briefly, exposure to several types of victimization was assessed repeatedly, using a standardized clinical interview protocol with mothers, when the children were 5, 7, 10, and 12 years of age and dossiers have been compiled for each child with cumulative information about exposure to physical and sexual abuse by an adult, emotional abuse and neglect, physical neglect, bullying by peers, and domestic violence. Exposure to each type of victimization was rated by coders as “0” not present; “1” probable harm, occasionally present, or evidence of only minor incidents (moderate abuse); or “2” definite harm, frequently present, or evidence of severe incidents (severe abuse).

*Childhood abuse/neglect* in this study included exposure to physical and sexual abuse by an adult, emotional abuse and neglect and physical neglect. In our study sample, 18.8% of the children experienced moderate abuse/neglect and 7.3% experienced severe abuse/neglect across childhood. A total of 36% have been exposed to occasional *bullying by peers* and 8.8% had been frequently bullied by peers. Finally, 28% were exposed to a single phase of *domestic violence* and 17.2% were exposed to repeated phases of domestic violence.

*Childhood poly-victimization* dossiers have been compiled for each child with cumulative information about exposure to physical abuse, sexual abuse, emotional abuse and neglect, physical neglect, bullying by peers and domestic violence. All childhood victimization experiences were summed: 1490 (73%) children had experienced no severe victimization; 423 (20.7%) had 1; 127 (6.3%) had 2 or more severe victimization experiences by age 12.

##### 2.2.1.2 Abuse/neglect, peer/sibling victimization and family violence in adolescence

These measures have been described previously (Fisher et al., 2015). In brief, at age 18, participants were interviewed about exposure to a range of adverse experiences between 12 and 18 years using the Juvenile Victimization Questionnaire 2nd revision (JVQ-R2) (Finkehor, Hamby, Turner, & Ormrod, 2011; Hamby, Finkelhor, Ormrod, & Turner, 2004), adapted as a clinical interview (Fisher et al., 2015). The JVQ has good psychometric properties (Finkehor, Hamby, Ormrod, & Turner, 2005; Fisher et al., 2015) and was used in the U.K. National Society for the Prevention of Cruelty to Children national survey (Radford et al., 2013; Radford et al., 2011), thereby providing benchmark values for comparisons with our cohort. Our adapted JVQ comprised 45 questions covering different forms of victimization grouped into seven categories: crime victimization, peer/sibling victimization, Internet/mobile phone victimization, sexual victimization, family violence, maltreatment, and neglect. All information from the adapted JVQ-R2 interview was compiled into victimization dossiers. Using these dossiers, each of the victimization categories was rated by trained researchers. Ratings were made using a 6-point scale: 0 = not exposed, then 1–5 for increasing levels of severity. The ratings for each type of victimization were then grouped into three classes: 0 – no exposure (score of 0), 1 – some (moderate) exposure (score of 1, 2 or 3), and 2 – severe exposure (score of 4 or 5) due to small numbers for some of the rating points.

For this study, *abuse/neglect in adolescence* included exposure to maltreatment, sexual victimization and neglect to match the variable of childhood abuse/neglect. In our study sample, 16.9% of the participants have experienced moderate (some) abuse/neglect in adolescence and 5.9% have experienced severe abuse/neglect in adolescence. A total of 42.4% have been exposed to some peer/sibling victimization and 15.3% have been exposed to severe peer/sibling victimization. Finally, 6.5% have been exposed to some family violence and 12% have been exposed to severe family violence.

*Adolescent poly-victimization.* Adolescent poly-victimization was derived by summing all seven victimization experiences coded as severe (“4” or “5”): 1321 (64.8%) adolescents had experienced no severe victimization; 391 (19.2%) had 1; 325 (15.9%) had 2 or more severe victimization experiences.

#### 2.2.2 ADHD

##### 2.2.2.1 Childhood ADHD

We ascertained ADHD diagnosis on the basis of mother and teacher reports of 18 symptoms of inattention and hyperactivity-impulsivity derived from DSM-IV (American Psychiatric Association (APA), 1994) diagnostic criteria and the Rutter Child Scales (Sclare, 1997). Participants had to have 6 or more symptoms reported by mothers or teachers in the past 6 months, with the other informant endorsing at least 2 symptoms. We considered participants to have a diagnosis of childhood ADHD if they met criteria at age 5, 7, 10, or 12. In total, 247 participants (12.1%; 71% of them boys) met criteria for ADHD across childhood: 6.8% at age 5, 5.4% at age 7, 3.4% at age 10 and 3.4% at age 12 years.

##### 2.2.2.2 Young adult ADHD

We ascertained ADHD at age 18 years based on private structured interviews with participants regarding 18 symptoms of inattention and hyperactivity-impulsivity according to DSM-5 criteria (American Psychiatric Association (APA), 2013). Participants had to endorse 5 or more inattentive and/or 5 or more hyperactivity-impulsivity symptoms to receive an ADHD diagnosis; we also required that symptoms interfered with individual’s “life at home or with family and friends” and “life at school or work” were rated 3 or higher on a scale (1, mild interference; 5, severe interference), thereby meeting criteria for impairment and pervasiveness. The DSM-5 (American Psychiatric Association (APA), 2013) requirement of symptom onset prior to age 12 was met if parents or teachers reported more than 2 ADHD symptoms at ages 5, 7, 10, or 12 years; a diagnosis of childhood ADHD was not required for young adult ADHD diagnosis. A total of 166 participants (8.1%) met criteria for ADHD at age 18, 52% of them male. Co-informants also rated participants on 8 ADHD symptoms, including 3 items relating to inattention and 5 items relating to hyperactivity/impulsivity.

### 2.3 Covariates

*Participants’ parental socio-economic status (SES)* was measured via a composite of parental income (total household), education (highest mother/father), and occupation (highest mother/father) when they were aged 5, and was categorized into tertiles (i.e., low-, medium-, and high-SES).

*IQ* at age 5 was measured using a short form of the Wechsler Preschool and Primary Scale of Intelligence—Revised (WPPSI–R; Wechsler, 1990). Using two subtests (Vocabulary and Block Design), children’s IQs were prorated following procedures described by Sattler (1992).

*IQ* at age 18 was measured using a short version of the Wechsler Adult Intelligence Scale–Fourth Edition (WAIS-IV; Wechsler, 2008). Using two subtests (Matrix Reasoning and Information), young adults’ IQs were prorated according to the method recommended by Sattler (2008).

*Mothers’ depression* was assessed using a modified version of the Diagnostic Interview Schedule (Robins, Cottler, Bucholz, & Compton, 1995). We assessed lifetime depression according to DSM-IV criteria (American Psychiatric Association (APA), 1994).

We derived a diagnosis of children’s *conduct disorder* on the basis of mothers’ and teachers’ reports on 14 of 15 items from DSM-IV (American Psychiatric Association (APA), 1994) criteria for conduct disorder (excluding “forced sexual activity” criteria, given the age of the participants). We considered participants to have a diagnosis of conduct disorder if they met five or more criteria at age 5, 7, 10, or 12. 15.6% of the children in the study sample met criteria for conduct disorder across childhood.

During the age-18 interview, we assessed participants’ *mental health* over the previous 12 months including depressive disorder, generalized anxiety disorder, post-traumatic stress disorder, alcohol dependence, cannabis dependence and *conduct disorder* according to DSM-IV (American Psychiatric Association (APA), 1994). Assessments were conducted in face-to-face interviews using the Diagnostic Interview Schedule (Robins et al., 1995). The assessment of *conduct disorder* was conducted as part of a computer-assisted module. A total of 38.8% of the young adults in this study sample experienced any of these mental health problems and a total of 14.9% had conduct disorders at age 18.

### 2.4 Statistical analysis

To examine the associations between abuse/neglect and ADHD diagnoses in childhood and in young adulthood, we used logistic regressions. We tested the robustness of our findings in three different ways. First, we used linear regressions to examine group differences between participants who experienced abuse/neglect and participants who did not on the ADHD total symptom scale and on inattentive and hyperactive/impulsive symptom sub-scales separately. Second, we controlled for potential childhood confounders including sex, IQ, parental SES and mother’s depression in logistic regression models. Third, for adult ADHD, we used linear regressions and repeated the analyses using a measure of ADHD symptoms reported by co-informants. We also examined whether associations with ADHD extended to: (1) other forms of victimization, including bullying and domestic violence; and (2) a cumulative measure of victimization (poly-victimization). We tested whether the association was concentrated among ADHD participants with comorbid conduct disorder, and additionally in young adulthood, with other forms of psychopathology. We further controlled for familial confounders by examining correlations between twins’ differences scores of poly-victimization and ADHD total symptom scale. For these analyses, we used continuous variables of exposure to violence and ADHD symptoms to maximise variation in both measures. We conducted the analyses with DZ and MZ twins together, and then only with MZ twins to control for all genetic confounding.

Regression analyses were conducted in Stata 14.1 (StataCorp, 2015). No interactions were found between sex and abuse/neglect in relation to ADHD in either childhood or young adulthood, therefore analyses were not stratified by gender. Participants in this study were pairs of same-sex twins, and each family contained data for two children, resulting in non-independent observations. To correct for this, we used tests based on the Huber-White or sandwich variance (Williams, 2000), which adjusts the estimated standard errors to account for the dependence in the data.

To examine the longitudinal associations between abuse/neglect and ADHD from childhood to young adulthood, we used structural equation modelling (SEM) procedures of Mplus 7.11 (Muthén & Muthén, 2012). We tested a full cross-lagged model with the autoregressive effects and both abuse/neglect and ADHD predicting each other at a later time point. This model accounted for the cross-sectional overlap and stability of variables. First, we conducted the analyses controlling for sex only. Second, we additionally controlled for age-5 IQ and parental SES. Third, we further controlled for conduct disorder in childhood. We accounted for non-independence of twin observations and non-normality of the data by using robust standard errors (Muthén & Muthén, 2012).

## 3 Results

### 3.1 Association between abuse/neglect and ADHD in childhood

**Fig. 1:**
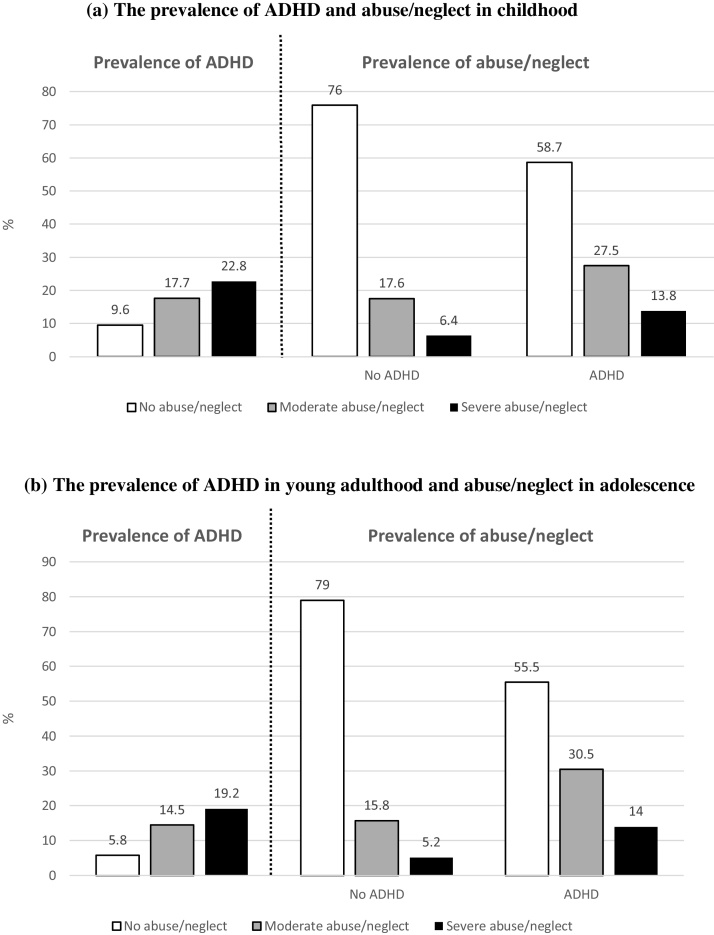
The prevalence of attention deficit hyperactivity disorder (ADHD) and abuse/neglect in (a) childhood, and (b) young adulthood.

Our findings indicate higher rates of children meeting diagnostic criteria for ADHD among those who were exposed to abuse/neglect compared to children who were not exposed to abuse/neglect (Fig. 1, panel a). Furthermore, higher rates of abuse/neglect were found among children with ADHD compared to those without ADHD diagnosis. Children exposed to moderate abuse/neglect had higher odds of 2.02 for meeting diagnostic criteria for ADHD compared to children who were not exposed, while children exposed to severe abuse/neglect had higher odds of 2.78 for having ADHD (Table 1). This association was robust to control for sex, age-5 IQ and parental SES, and became marginal when controlling for mother’s depression. However, the association remained significant when we merged the two groups of children who experienced moderate and severe abuse/neglect. We replicated this association using a total scale of ADHD symptoms (Table 2). Group differences were similar when we separately examined inattentive and hyperactive/impulsive symptom sub-scales.

**Table 1:** Associations between victimization and ADHD in childhood.

	Unadjusted	Adjusted for sex, IQ and SES	Adjusted for sex, IQ, SES and mother’s depression
	OR (95% CI)	OR (95% CI)	OR (95% CI)
**Abuse/neglect**			
Moderate	2.02 (1.43–2.86)***	1.55 (1.07–2.26)*	1.47 (1.00–2.15)
Severe	2.78 (1.72–4.48)***	1.72 (1.05–2.83)*	1.60 (0.97–2.64)
Any	1.75 (1.42–2.16)***	1.37 (1.09–1.71)*	1.31 (1.05–1.65)*

**Bullying by peers**	5.22 (3.44–7.90)***	3.98 (2.52–6.27)***	3.70 (2.32–5.90)***
**Domestic violence**	2.33 (1.59–3.43)***	1.71 (1.13–2.58)*	1.54 (1.01–2.36)*

**Poly-victimization**			
Exposure to one type of victimization	2.20 (1.57–3.07)***	1.83 (1.26–2.64)**	1.75 (1.20–2.55)**
Exposure to two or more types of victimization	3.97 (2.46–6.42)***	2.50 (1.52–4.12)***	2.31 (1.39–3.84)**

*Note*. ADHD, attention deficit hyperactivity disorder. CI, confidence intervals. IQ, intelligence quotient. OR, odds ratio. SES, socio-economic status.

* p < 0.05.

** p < 0.01.

*** p < 0.001.

**Table 2:** Childhood ADHD symptom scores by abuse/neglect status in childhood.

	No abuse/neglect (n = 1507)	Moderate abuse/neglect (n = 384)			Severe Abuse/neglect (n = 149)		
	M (SD)	M (SD)	t	Cohen’s d	M (SD)	t	Cohen’s d
**Total ADHD scale**	2.68 (0.09)	4.89 (0.24)	5.91***	0.59	6.49 (0.45)	5.54***	1.03
**Inattentive sub-scale**	1.13 (0.05)	1.99 (0.12)	4.43***	0.45	2.88 (0.25)	4.58***	0.91
**Hyperactive/impulsive sub-scale**	1.55 (0.05)	2.88 (0.14)	6.39***	0.62	3.62 (0.23)	5.97***	1.00

*Note*. ADHD, attention deficit hyperactivity disorder. M, mean. SD, standard deviation.

*** p < 0.001.

The association between abuse/neglect and ADHD in childhood extended to other forms of childhood victimization: children who were frequently bullied or were exposed to repeated phases of domestic violence had greater odds for ADHD diagnosis (Table 1). Furthermore, we found that childhood ADHD was associated with being exposed to poly-victimization: children who were exposed to more than one type of victimization had higher odds for having a diagnosis of ADHD (Table 1).

ADHD was highly comorbid with conduct disorder in our sample: 118 (47.8%) children with ADHD had comorbid conduct disorder. Prevalence of exposure to abuse/neglect among sub-groups of children with ADHD, with or without comorbid conduct disorder, is presented in Fig. S1 (panel a). We found that the risk for being exposed to abuse/neglect was concentrated among children with ADHD and comorbid conduct disorder (Table S1).

### 3.2 Association between abuse/neglect and ADHD in young adulthood

Similar to childhood, we found an over-representation of young adults with ADHD among those who were exposed to abuse/neglect between 12–18 years, as well as an over-representation of those who experienced abuse/neglect among young adults with ADHD (Fig. 1, panel b). Young adults who were exposed to moderate abuse/neglect during adolescence had higher odds of 2.76 for ADHD compared to those who were not victimized (Table 3). In addition, young adults who were exposed to severe abuse/neglect in adolescence had higher odds of 3.86 for ADHD. The association between abuse/neglect and ADHD diagnosis was robust to control for confounders including sex, age-18 IQ and parental SES. We replicated the association between adult ADHD and abuse/neglect in adolescence using a total scale of ADHD symptoms (Table 4). We observed similar group differences when examining separately sub-scales of inattentive and hyperactive/impulsive symptoms. Furthermore, the association between abuse/neglect and ADHD was not simply an artefact of using self-reports of ADHD in young adulthood: findings indicated that those who were exposed to moderate abuse/neglect had more ADHD symptoms according to co-informants’ reports (M = 0.78, SD = 0.09) compared to those without ADHD (M = 0.50, SD = 0.03; Cohen’s d = 0.21). We observed a similar finding for those who experienced severe abuse/neglect (M = 1.17, SD = 0.18; Cohen’s d = 0.50).

**Table 3:** Associations between victimization in adolescence and ADHD in young adulthood.

	Unadjusted	Adjusted for sex, IQ and SES
	OR (95% CI)	OR (95% CI)
**Abuse/neglect**		
Moderate	2.76 (1.94–3.93)***	2.93 (2.03–4.22)***
Severe	3.86 (2.33–6.39)***	3.59 (2.10–6.14)***

**Bullying by peers**	2.71 (1.71–4.29)***	2.69 (1.69–4.29)***
**Domestic violence**	2.27 (1.50–3.44)***	2.12 (1.37–3.26)**

**Poly-victimization**		
Exposure to one type of victimization	1.98 (1.32–2.97)**	1.79 (1.18–2.74)**
Exposure to two or more types of victimization	3.30 (2.24–4.88)***	3.05 (2.03–4.57)***

*Note*. ADHD, attention deficit hyperactivity disorder. CI, confidence intervals. IQ, intelligence quotient. OR, odds ratio.

SES, socio-economic status.

** p < 0.01.

*** p < 0.001.

**Table 4:** Young adult ADHD symptom scores by abuse/neglect in adolescence.

	No abuse/neglect (n = 1573)	Moderate abuse/neglect (n = 345)			Severe Abuse/neglect (n = 120)		
	M (SD)	M (SD)	t	Cohen’s d	M (SD)	t	Cohen’s d
**Total ADHD scale**	5.22 (0.10)	7.24 (0.22)	7.90***	0.49	8.41 (0.40)	6.63***	0.77
**Inattentive sub-scale**	2.74 (0.06)	3.70 (0.13)	6.57***	0.41	4.11 (0.22)	5.19***	0.59
**Hyperactive/impulsive sub-scale**	2.48 (0.06)	3.53 (0.13)	7.48***	0.46	4.30 (0.24)	6.55***	0.78

*Note*. ADHD, attention deficit hyperactivity disorder. M, mean. SD, standard deviation.

*** p < 0.001.

As in childhood, the association between abuse/neglect in adolescence and young adult ADHD extended to other forms of victimization: participants who reported being severely victimized by peers or being exposed to severe family violence in adolescence had increased odds for young adult ADHD (Table 3). Furthermore, we found that adult ADHD was associated with being exposed to multiple types of victimization in adolescence: young adults who were exposed to more than one type of victimization in adolescence had higher odds to have ADHD (Table 3).

Similar to childhood, young adults with ADHD often had comorbid conduct disorder (36.2%), but also other forms of psychopathology (68.7%). Prevalence rates of exposure to abuse/neglect among sub-groups of young adults with a diagnosis of ADHD, with or without comorbidity, are presented in Fig. S1 (panel b and c). We found that the odds for moderate and severe abuse/neglect in adolescence were elevated among adults with ADHD and comorbid conduct disorder, as were the odds among those with ADHD alone (Table S1). We found similar elevated odds of abuse/neglect among the adults with ADHD and other forms of psychopathology, while the odds decreased but remained significant for those with ADHD only.

### 3.3 Controlling for familial confounding

We examined differences between twins on poly-victimization and ADHD symptoms. In childhood, we found a modest association between twins’ difference scores on poly-victimization and difference scores on ADHD total symptoms scale (r = 0.13, p < 0.001). This indicates that within a twin pair, the twin who had higher score on poly-victimization also had more ADHD symptoms. This association became not significant when repeated with MZ twins only (r = 0.07, p = 0.101), indicating that the association between poly-victimization and ADHD symptoms in childhood was accounted for by genetic factors. In young adulthood, we found a modest association between twins’ difference scores on poly-victimization and difference scores on ADHD symptoms (r = 0.18, p < 0.001). This association remained when repeated with MZ twins only, thus controlling for shared environment as well as genetic factors (r = 0.17, p < 0.001). This finding indicates that the association between poly-victimization and ADHD symptoms in young adulthood is partly environmentally-driven.

### 3.4 Longitudinal associations between abuse/neglect and ADHD from childhood to young adulthood

**Fig. 2:**
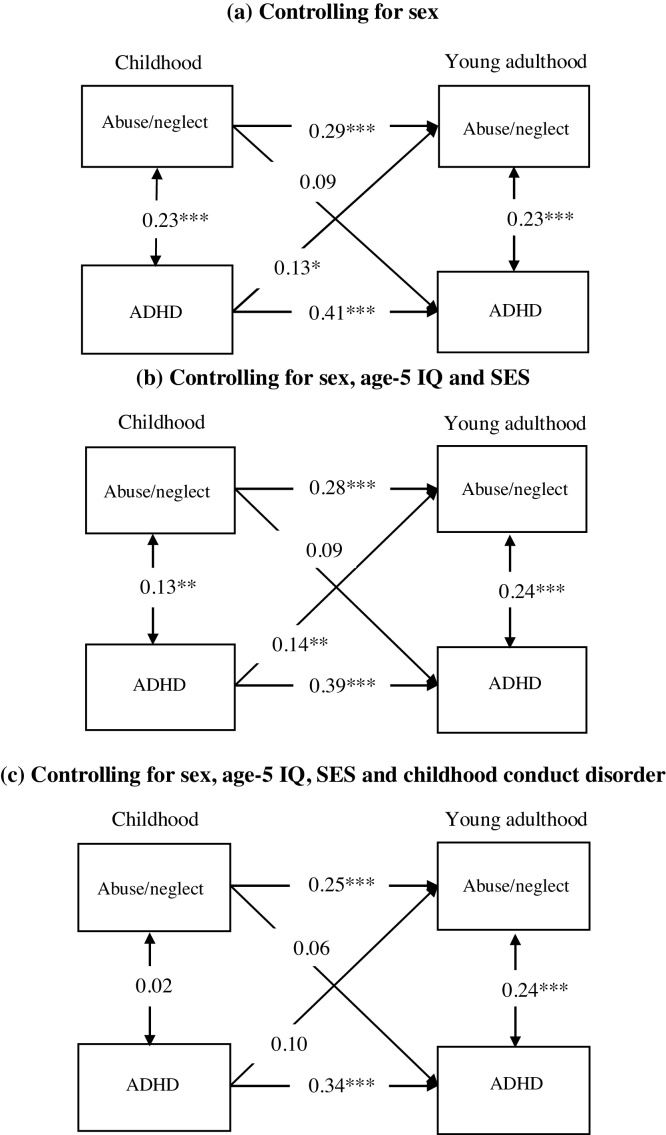
Longitudinal associations between abuse/neglect and attention deficit hyperactivity disorder (ADHD) from childhood to young adulthood, when controlling for (a) sex, (b) sex, age-5 IQ, and parental socio-economic status (SES), and (c) sex, age-5 IQ, SES and childhood conduct disorder. All associations are expressed as standardized path coefficients. *p < 0.05, **p < 0.01, ***p < 0.001.

Abuse/neglect in childhood was not associated with ADHD in young adulthood taking into account sex (*B* = 0.09, *p* = 0.115; Fig. 2, panel a). However, we found that childhood ADHD was associated with abuse/neglect in later years (*B* = 0.13, *p* = 0.011). This longitudinal association was robust to adjustment for the stability of being exposed to abuse/neglect up to age 18, for ADHD from childhood to young adulthood, and also for concurrent associations between abuse/neglect and ADHD in childhood and in young adulthood. When we controlled for age-5 IQ and parental SES (Fig. 2, panel b), the association between childhood ADHD and abuse/neglect in adolescence remained significant (*B* = 0.14, *p* = 0.013). When we further controlled for conduct disorder in childhood (Fig. 2, panel c), the association between childhood ADHD and later abuse/neglect became not significant (*B* = 0.10, *p* = 0.111). This finding indicates that the longitudinal association between ADHD and later abuse/neglect is specific to those participants with comorbid conduct disorder in childhood.

## 4 Discussion

Our study using data from a prospective cohort of twins provides three notable findings on the associations between abuse/neglect and ADHD. First, concurrent analyses showed that abuse/neglect was strongly and robustly associated with ADHD in childhood, but also in young adulthood, indicating that this known link is not limited to childhood years. These associations survived control for SES, IQ, shared environmental and genetic confounds and extended to other forms of victimization, but in childhood, was concentrated among children with ADHD and comorbid conduct disorder. Second, longitudinal analyses indicated that childhood abuse/neglect did not predict later ADHD. This finding is contrary to previous studies using global retrospective measures of maltreatment up to young adulthood. Third, childhood ADHD was associated with later exposure to abuse/neglect when comorbid with conduct disorder. This indicates that disruptive behaviors, and not ADHD symptoms per se, have a long-term influence on the way the environment responds to individuals. Our findings shed a new light on the longitudinal associations between ADHD and maltreatment, calling for replications of these findings.

### 4.1 ADHD as a risk factor for later abuse/neglect

Children’s mental health symptomatology increases their risk of maltreatment, peer victimization and sexual victimization (Turner, Finkelhor, & Ormrod, 2010). Our findings are in line with previous studies showing that disruptive behaviors, including ADHD and conduct disorder, may increase future risk of exposure to abuse and neglect. Symptoms associated with ADHD and conduct disorder - including aggressiveness, impulsiveness and noncompliance - may pose caregiving challenges and make children vulnerable to various forms of victimization in childhood (Sari Gokten et al., 2016). Our findings extend others’ findings by showing that ADHD increased risk for later abuse/neglect in adolescence. At least two hypotheses may explain this result. Firstly, this association could be accounted for by the continuation of ADHD symptoms and conduct problems into the adult years. While this hypothesis can partly account for this longitudinal association, it cannot explain it completely as we found the association to be significant over and above ADHD symptoms at age 18, indicating that young adults with remitted ADHD are nevertheless at risk for experiencing abuse/neglect. Secondly, ADHD and conduct problems may have a long-lasting influence on relationships. Despite ADHD symptoms having remitted, it is possible that others’ presumptions about one’s behaviors are what preserve the pattern of relationships that are difficult to change in later life. Our findings add support to the growing body of evidence suggesting that children’s temperament and behavior influence the response and reaction of others towards them (Danese et al., 2017; Gault-Sherman, 2012; Kerr & Stattin, 2003; Pardini, 2008; Wertz et al., 2016). They also emphasize an important role for preventative monitoring of children with ADHD and conduct problems to reduce their risk for harm as parents may struggle to cope with children’s behaviors and demands, possibly influencing children’s risk for experiencing adversity. Close monitoring of this risk should be included as part of routine assessment with health professionals. Future research should examine the role of possible mediators, such as parenting skills and distress tolerance.

### 4.2 Lack of support for child abuse/neglect causing ADHD

Our findings do not support previous conclusions that childhood maltreatment is an environmental risk factor for ADHD in adulthood (Capusan et al., 2016). Nevertheless, we cannot completely rule out the possibility that exposure to abuse/neglect can increase vulnerability to developing ADHD symptoms, as reported recently (Dinkler et al., 2017). Previous studies have generated findings linking biological disruptions associated with adverse childhood experiences, including maltreatment, to greater risk for a variety of chronic diseases well into the adult years (Shonkoff et al., 2012). There is growing evidence for the extent to which both the cumulative burden of stress over time (e.g., from chronic maltreatment) and the timing of specific environmental insults during sensitive developmental periods can create structural and functional disruptions that lead to a wide range of physical and mental illnesses later in adult life (Shonkoff et al., 2012). However, as our findings do not support a causal link between abuse/neglect and ADHD, we suggest a careful interpretation of findings that may suggest that child maltreatment causes ADHD.

### 4.3 Abuse/neglect and ADHD in young adulthood

For the first time, we found strong associations between abuse/neglect in adolescence and ADHD in young adulthood. These associations are robust to control for potential confounders and using ADHD symptoms scales and informant reports. Nonetheless, we found these associations to be nonspecific, as they extended to other forms of victimization. Our findings are consistent with previous studies demonstrating that adult mental health is similarly influenced by a wide range of adverse exposures (Schaefer et al., 2017; Vachon, Krueger, Rogosch, & Cicchetti, 2015). Different from childhood, we found that this association in young adulthood is not accounted for by other behavioral or psychiatric disorders. This is despite the high prevalence of comorbidity. This suggests differences between ADHD in childhood and in adulthood, and points to the need for further studies to explore the unique features of adult ADHD and its predictors. We also found that this association in young adulthood is environmentally-driven. This can be explained by the process of gaining more independence during these years, and the new interactions with people outside the family and the education system. Our findings highlight the importance of taking into consideration victimization in adolescence and examining its consequences. Furthermore, the assessment of adolescents and young adults with ADHD should include inquiry about exposure to victimization in adolescence in addition to the childhood years.

### 4.4 Limitations

The strength of our study includes the use of prospective as well as repeated measures of both abuse/neglect and ADHD up to young adulthood in a nationally-representative cohort. However, our findings should be considered in light of some limitations. First, the assessment of victimization in adolescence covered a longer period of time compared to young adult ADHD which covered symptoms in the past year. However, participants were interviewed face-to-face using a well-established measure and the assessment referred to a specific time-frame (i.e., secondary school). In addition, referring to this time-period enabled us to gather detailed information regarding the exposure to victimization throughout adolescence. Second, due to our relatively small sample size, we had limited statistical power when looking at twins’ differences among our group of MZ twins. A larger sample size would facilitate further examination of twins’ differences in twin pairs discordant for abuse/neglect. Third, the E-Risk sample is composed of twins, so the results may not generalize to singletons. Reassuringly, the prevalence of childhood abuse/neglect as well as the prevalence rates of victimization exposure between 12 and 18 years in our sample matches recent UK general population estimates (Radford et al., 2013; Radford et al., 2011). The prevalence of childhood ADHD at each age in our sample is well within the range of 3.4%–11% estimated previously (Polanczyk et al., 2015) and our rate of ADHD persistence is similar to that found in a meta-analysis (Faraone et al., 2006).

### 4.5 Implications for research and clinical practice

We provided additional evidence regarding the robustness of the associations between maltreatment and ADHD, emphasising the important role of comorbid conduct disorder. We also showed that this association is not limited to childhood and not specific to abuse/neglect. Our findings highlight the possibility of a long-term effect of disruptive behavior on the risk of experiencing violence victimization, rather than the other way around. Although our study does not support previous causal inferences regarding the relationship between child maltreatment leading to adult ADHD, it emphasises the complexity of establishing causality. Additional research using prospective longitudinal designs is important to examine whether our findings can be replicated. Another factor to be considered when examining the direction of the association from ADHD to abuse/neglect is the presence of ADHD symptoms among the parents of a child with ADHD. Since ADHD is a heritable condition (Thapar & Cooper, 2016; Thapar et al., 2013), it is probable that at least one of the parents of a child with ADHD also experience similar symptoms. This adds to the complexity of parent-child relationships. Further research is needed to examine to what extent parents’ ADHD symptoms influence their parenting, especially with a child with ADHD.

Our study also has clinical implications. First, our findings emphasize that clinicians treating people with ADHD, and especially those with comorbid conduct disorder, should be aware that their patients are at heightened risk for current and future maltreatment and of other forms of violence victimization. This indicates the importance of conducting an evaluation of concurrent and past victimization during routine assessment and treatment planning of people with ADHD. Second, our findings suggest that along with interventions focusing on children’s ADHD and conduct disorder symptoms there is a need to provide guidance and support to carers. Knowledge about behavioral problems might help to better understand the potential challenges they are facing. Teaching them various strategies that can be used in order to facilitate behavior and function might give them more effective ways to deal with the child’s behavior. Third, our findings suggest that while maltreatment may not directly cause ADHD, maltreatment and ADHD are associated and mental health professionals and clinical services that are in contact with children, adolescents and adults who experienced maltreatment should be aware of their higher risk of having ADHD.

## Funding

The E-Risk Study is funded by the Medical Research Council (UKMRC grant G1002190). Additional support was provided by National Institute of Child Health and Human Development (grant HD061298), National Society for Prevention of Cruelty to Children (NSPCC) and Economic and Social Research Council (ESRC), The Avielle Foundation, and by the Jacobs Foundation. Adi Stern is supported by The Haruv Institute’s Post-Doctoral Students Fellowship and by the Humanitarian Trust Fellowship. Helen L. Fisher is supported by an MQ Fellows Award (MQ14F40). Louise Arseneault is the Mental Health Leadership Fellow for the UK Economic and Social Research Council (ESRC).
