# Supplementary material for: Associations between abuse/neglect and ADHD from childhood to young adulthood: A prospective nationally-representative twin study
Source: Child Abuse Negl. 2018 Jul;81:274–85. doi: 10.1016/j.chiabu.2018.04.025 (PMC6013278; doi:10.1016/j.chiabu.2018.04.025)
Supplement: Supplementary file 1 [file mmc1.docx]

**Supplementary Materials to Stern et al., Associations between abuse/neglect and ADHD from childhood to young adulthood: A prospective nationally-representative twin study**

These Supplementary Materials contain additional details about the measurement of victimization experiences in childhood. Furthermore, they contain supplemental figures and tables to accompany statistical analyses reported in the Main Article.

*Assessment of childhood victimization*

Exposure to several types of abuse/neglect was assessed repeatedly when the children were 5, 7, 10, and 12 years of age and dossiers were compiled for each child with cumulative information about exposure to physical and sexual abuse by an adult, emotional abuse and neglect, physical neglect, bullying by peers and domestic violence. All the component measures are outlined briefly below.

*Physical and sexual abuse by an adult.* When the twins were aged 5, 7, 10 and 12 years of age their mothers were interviewed about each twins’ experience of intentional harm by an adult. An unusual feature of our assessments is that we repeatedly interviewed mothers over the years, allowing them to build confidence in the research team. Also, we were able to reassure mothers that if harm to the child was ongoing and had to be reported by us, reporting would be managed through a trusted person, namely the family’s registered GP. As the children got older, some mothers who were initially reluctant to reveal abuse, revealed details of severe abuse at a later interview. At age 5 we used the standardized clinical protocol from the MultiSite Child Development Project (Dodge, Bates, & Pettit, 1990; Lansford et al., 2002). At ages 7, 10, and 12 this interview was modified to expand its coverage of contexts for child harm. Interviews were designed to enhance mothers’ comfort with reporting valid child maltreatment information, while also meeting researchers’ responsibilities for referral under the U.K. Children Act. Specifically, mothers were asked whether either of their twins had been intentionally harmed (physically or sexually) by an adult or had contact with welfare agencies. When mothers reported any maltreatment, interviewers followed with standardized probes (e.g., accidental harm was ruled out; harm by age peers was coded as bullying, not maltreatment). Sexual abuse was queried directly. Under the U.K. Children Act, our responsibility was to secure intervention if maltreatment was current and ongoing. Such intervention on behalf of E-Risk families was carried out with parental cooperation in all but one case. No family has left the study after intervention. Over the years of data collection, the study maintained a cumulative profile for each child, composed of the caregiver reports, recorded debriefings with interviewers who had coded any indication of maltreatment at any of the four successive home visits, recorded narratives of the four successive caregiver interviews (covering the period from birth to 12 years), and information from clinicians whenever the Study team made a child-protection referral. At the end of the age–12 phase, based on review of each child’s cumulative dossier, two clinical psychologists reached consensus for whether physical maltreatment or sexual abuse had occurred. Inter-rater agreement between the coders was 90% of cases for whom maltreatment was identified (100% for cases of sexual abuse), and discrepantly coded cases were resolved by consensus review. These were coded as: 0 = no physical or sexual abuse at any age; 1 = probable physical or sexual abuse at any age; and 2 = definite physical or sexual abuse at any age.

*Emotional abuse and neglect*. These forms of maltreatment were coded from research workers’ narratives of the home visits at ages 5, 7, 10, and 12. We coded quite severe examples of parental behavior observed. For example, a mother who had schizophrenia screamed and swore at the children throughout the home visit. As another example, a father who was drunk during the home visit repeatedly spoke abusively to the children in front of the research workers. We found that coders could not empirically separate emotional abuse and emotional neglect in a reliable way and thus such experiences were coded together as emotional abuse/neglect. Inter-rater agreement between the coders exceeded 85% for cases with emotional abuse and neglect, and discrepant cases were resolved by consensus review. Children with no evidence of emotional abuse/neglect were coded as 0, those where there was some indication of emotionally inappropriate/potentially abusive or neglectful behavior were coded as 1, and where there was evidence of severe emotional abuse/neglect the children were coded as 2.

*Physical neglect*. The cumulative observations of the physical state of the home environment documented by the research workers during home visits to the twins at ages 5, 7, 10 and 12 were reviewed by two raters for evidence of physical neglect. This was defined as any sign that the caretaker was not providing a safe, sanitary, or healthy environment for the child. This included the child not having proper clothing or food, as well as grossly unsanitary home environments. (However, this did not include a family living in a crime-ridden neighbourhood for economic reasons). Inter-rater agreement between the coders was 85%, and discrepantly coded cases were resolved by consensus review. Children with no evidence of physical neglect were coded as 0, those for whom there was an indication of minor physical neglect were coded as 1, and where there was evidence of severe physical neglect the children were coded as 2.

*Bullying by peers.* We assessed experiences of victimization by bullies using both mothers’ and children’s reports. During the interview, the following standard definition of bullying was read out: “someone is being bullied when another child (a) says mean and hurtful things, makes fun, or calls a person mean and hurtful names; (b) completely ignores or excludes someone from their group of friends or leaves them out on purpose; (c) hits, kicks, or shoves a person, or locks them in a room; (d) tells lies or spreads rumours about them; and (e) other hurtful things like these. We call it bullying when these things happen often, and when it is difficult to make it stop. We do not call it bullying when it is done in a friendly or playful way”. Mothers were interviewed when children were 7, 10, and 12 years old and asked whether either twin had been bullied by another child, responding never, yes, or frequently. We combined mothers’ reports at child age 7 and 10 to derive a measure of victimization during primary school.

Mothers’ reports when the children were 12 years old indexed victimization during secondary school. During private interviews with the children when they were 12 years old, the children indicated whether they had been bullied by another child during primary or secondary school. When a mother or a child reported victimization, the interviewer asked them to describe what happened. Notes taken by the interviewers were later checked by an independent rater to verify that the events reported could be classified as instances of bullying operationally defined as evidence of (a) repeated harmful actions, (b) between children, and (c) where there is a power differential between the bully and the victim. Although inter-rater reliability between mothers and children was only modest (kappa = 0.20–0.29), reports of victimization from both informants were similarly associated with children’s emotional and behavioral problems, suggesting that each informant provides a unique but meaningful perspective on bullying involvement (Shakoor et al., 2011). We thus combined mother and child reports of victimization to capture all instances of bullying victimization for primary and secondary school separately: reported as not victimized by both mother and child; reported by either mother or child as being occasionally victimized; and reported as being occasionally victimized by both informants or as frequently victimized by either mother or child or both (Bowes et al., 2013). We then combined these primary and secondary school ratings to create a bullying victimization variable for the entire childhood period (5–12 years). Children who were never bullied in primary or secondary school or occasionally bullied during one of these time periods were coded as 0; children who were occasionally bullied during primary and secondary school, or frequently bullied during one of these time periods were coded as 1; and children who were frequently bullied at both primary and secondary school were coded as 2.

*Domestic violence.* Mothers reported about perpetration by and victimization of 12 forms of physical violence (e.g., slapping, hitting, kicking, strangling) from the Conflict Tactics Scale (Moffitt, Caspi, Krueger, Magdol, & et al., 1997; Straus & Gelles, 1990), on three assessment occasions during the child’s first decade of life (when the children were 5, 7, and 10 years of age). Reports of either perpetration or victimization constituted evidence of domestic violence. Families in which no physical violence took place were coded as 0; families in which physical violence took place on one occasion were coded as 1; and families in which physical violence took place on multiple occasions were coded as 2.

Moderate

35.6%

1. **ADHD and comorbid psychopathology in young adulthood**

**(b) ADHD and comorbid conduct disorder in young adulthood**

**Abuse/neglect**

**ADHD**

Moderate

34.3%

Moderate

20.2%

**Conduct disorder**

**ADHD and**

**conduct disorder**

**Abuse/neglect**

**Abuse/neglect**

Severe

7.0%

Severe

21.2%

Severe

20.9%

**Abuse/neglect**

**ADHD**

Moderate

24.5%

Moderate

26.0%

**Conduct disorder**

**ADHD and**

**conduct disorder**

**Abuse/neglect**

**Abuse/neglect**

Severe

15.4%

Severe

11.9%

Severe

14.7%

Moderate

39.0%

**Abuse/neglect**

**ADHD**

Moderate

24.9%

Moderate

21.2%

**Psychopathology**

**ADHD and**

**psychopathology**

**Abuse/neglect**

**Abuse/neglect**

Severe

5.8%

Severe

17.9%

Severe

11.8%

Moderate

34.8%

**(a) ADHD and comorbid conduct disorder in childhood**

**Fig. S1.** The prevalence of abuse/neglect among sub-groups of participants with attention deficit hyperactivity disorder (ADHD) and comorbid (a) conduct disorder in childhood,

(b) conduct disorder young adulthood, and (c) psychopathology in young adulthood

**Table S1**. Associations between abuse/neglect and ADHD with and without comorbid conditions in childhood and in young adulthood

|  | **Moderate abuse/neglect**  Adjusted OR (95% CI) | **Severe abuse/neglect**  Adjusted OR (95% CI) |
| --- | --- | --- |
| **Childhood** |  |  |
| ADHD without comorbid conduct disorder | 1.20 (0.71-2.01) | 1.32 (0.63-2.80) |
| ADHD and comorbid conduct disorder | 2.19 (1.34-3.59)** | 3.21 (1.71-6.04)*** |
| **Young adulthood** |  |  |
| ADHD without comorbid conduct disorder | 2.20 (1.38-3.51)** | 4.25 (2.30-7.88)*** |
| ADHD and comorbid conduct disorder | 4.64 (2.59-8.30)*** | 3.38 (1.46-7.86)** |
| ADHD without comorbid psychopathology | 2.48 (1.23-5.00)* | 4.11 (1.12-15.19)* |
| ADHD and comorbid psychopathology | 4.97 (3.15-7.84)*** | 13.32 (6.53-27.20)*** |

Note. ADHD, attention deficit hyperactivity disorder. CI, confidence intervals. OR, odds ratio. *p<0.05, **p<0.01, ***p<0.001

**References**

Bowes, L., Maughan, B., Ball, H., Shakoor, S., Ouellet-Morin, I., Caspi, A., … Arseneault, L. (2013). Chronic bullying victimization across school transitions: The role of genetic and environmental influences. *Development and Psychopathology, 25*(2), 333-346.

Dodge, K. A., Bates, J. E., & Pettit, G. S. (1990). Mechanisms in the cycle of violence. Science, 250, 1678+.

Lansford, J.E., Dodge, K.A., Pettit, G.S., Bates, J.E., Crozier, J., & Kaplow, J. (2002). A 12-year prospective study of the long-term effects of early child physical maltreatment on psychological, behavioral, and academic problems in adolescence. *Archives of Pediatrics & Adolescent Medicine, 156(8)*, 824–830.

Moffitt, T. E., Caspi, A., Krueger, R. F., Magdol, L., & et al. (1997). Do partners agree about abuse in their relationship?: A psychometric evaluation of interpartner agreement. *Psychological Assessment, 9*(1), 47-56.

[Shakoor](file:///C:\Users\k1634363\Desktop\HTML\Refs\E-Risk%20Papers\Shakoor%20et%20al%202011.pdf), S., Jaffee, S.R., Andreou, P., Bowes, L., Ambler, A.P., Caspi, A., Mofitt, T.E., & Arseneault, L. (2011). Mothers and children as informants of bullying victimization: Results from an epidemiological cohort of children. *Journal of Abnormal Child Psychology, 39*(3), 379-387.

Straus, M. A., & Gelles, R. J. (1990). *Physical violence in American families: Risk factors and adaptations to violence in 8,145 families*. New Brunswick, NJ: Transaction Publishing.
